# Supplementary material for: The Dynamic Change in Aromatic Compounds and Their Relationship with CsAAAT Genes during the Post-Harvest Process of Oolong Tea
Source: Metabolites. 2023 Jul 20;13(7):868. doi: 10.3390/metabo13070868 (PMC10385818; doi:10.3390/metabo13070868)
Supplement: Supplementary file 1 [file metabolites-13-00868-s001.zip › Figure S1.pdf]

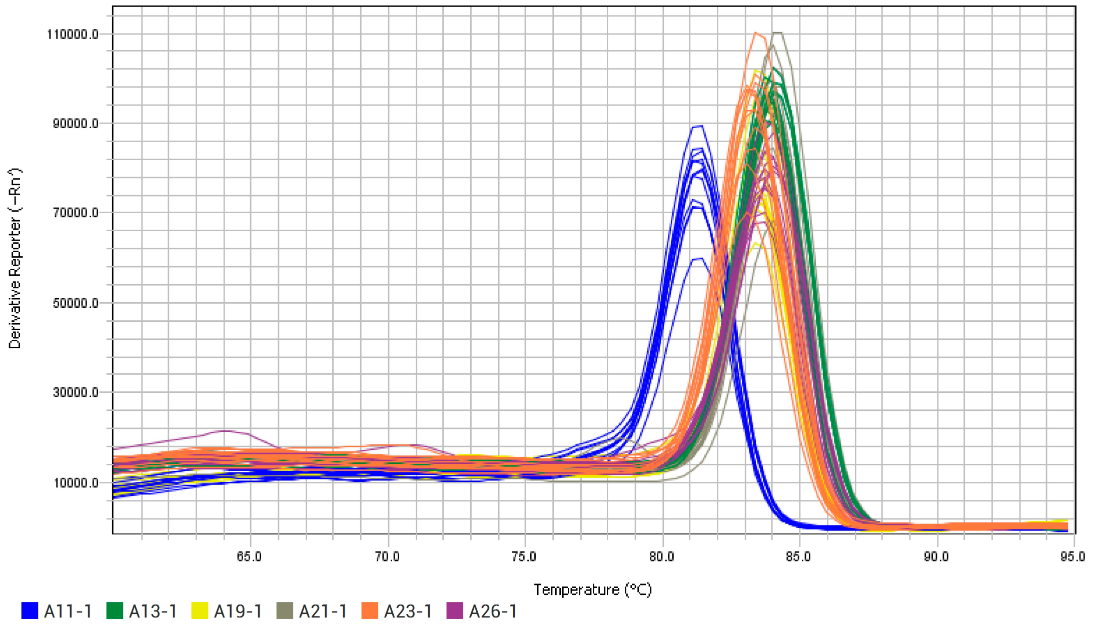

Figure S1 The melting curve plot of key *CsAAAT* genes during the post-harvest process of oolong tea

The specific melting curve pattern was used to validate the FPKM of key CsAAAT genes. The X-axis showed the The X-axis represented the process of heating up(range from 60℃ to 95 ℃). The y-axis showed the derivative reporter(-Rn). A11-1, A13-1, A19-1, A21-1, A23-1, and A26-1 represented *CsAAAT1-1, CsAAAT1-3, CsAAAT1-9, CsAAAT2-1, CsAAAT2-3, CsAAAT2-6* gene, respectively.
