# Supplementary material for: The Dynamic Change in Aromatic Compounds and Their Relationship with CsAAAT Genes during the Post-Harvest Process of Oolong Tea
Source: Metabolites. 2023 Jul 20;13(7):868. doi: 10.3390/metabo13070868 (PMC10385818; doi:10.3390/metabo13070868)
Supplement: Supplementary file 1 [file metabolites-13-00868-s001.zip › Figure S2.pdf]

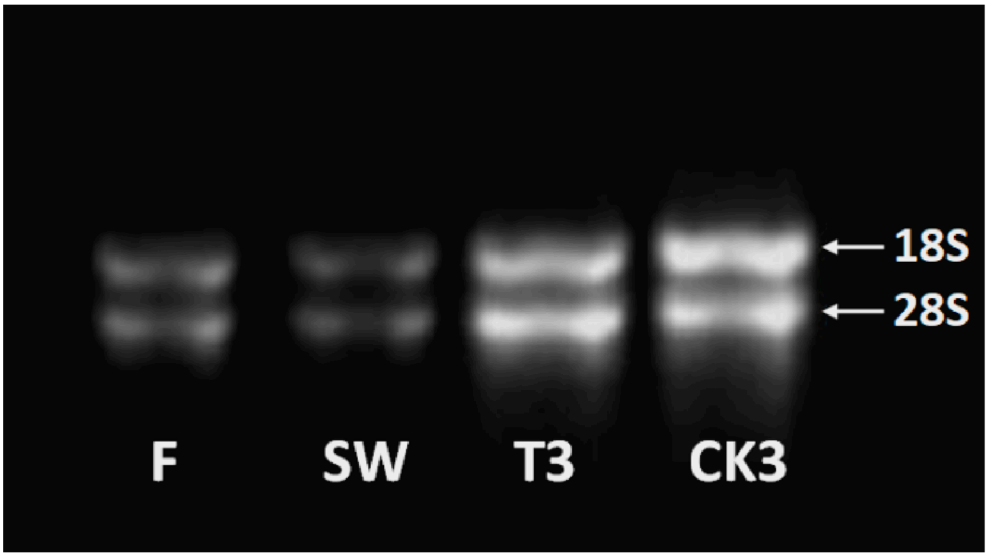

Figure S2 The electrophoretogram of total RNA of tea leaf samples during the post-harvest processing steps

F: fresh tea leaves; SW: solar-withered leaves; T3: turn-over treatment for three times; CK3: control group without turn-over treatment for three times.

The clear 18S bands and 28S bands of tea leaf samples could be distinguished, which indicated the purity of total RNA of each sample was good
