# Supplementary material for: The Dynamic Change in Aromatic Compounds and Their Relationship with CsAAAT Genes during the Post-Harvest Process of Oolong Tea
Source: Metabolites. 2023 Jul 20;13(7):868. doi: 10.3390/metabo13070868 (PMC10385818; doi:10.3390/metabo13070868)
Supplement: Supplementary file 1 [file metabolites-13-00868-s001.zip › Table S1.pdf]

Table S1 The specific primers for qRT-PCR

Gene symbol	Forward primer sequence (5′–3′)	Reverse primer sequence (5′–3′)	Melting temperature(°C)	Amplicon length (bp)
CsAAAT1-1	GCAGACCATACCATACCAGAG	ATGTTCGTCGGCGTTTC	57.2/58.1	119
CsAAAT1-3	CACAATCAAAGGCATTCTGG	TTCTTCAGCAACAACAGTGG	59.1/57.4	130
CsAAAT1-9	CCTCCTCAAAGACATTAGCG	AAAGACTTCACCCTCCCGA	57.6/59.6	168
CsAAAT2-1	AACAGTTCTTGCTCGTCCA	ACCTCCCAACCTTTCTCTG	56.9/56.7	132
CsAAAT2-3	TTCTGCTCCCAAGACCA	ACCTCCCAACCTTTCTCTG	56.0/56.7	103
CsAAAT2-6	TCTCATACACACACACACACG	AGCCCTTTGTTCCCTTG	56.4/56.1	107
CsGAPDH	TTGTCATCGTTGAGGGTCT	CAGTGGGAACGCGGAAAGC	61.6/61.7	206
