# Supplementary material for: The Dynamic Change in Aromatic Compounds and Their Relationship with CsAAAT Genes during the Post-Harvest Process of Oolong Tea
Source: Metabolites. 2023 Jul 20;13(7):868. doi: 10.3390/metabo13070868 (PMC10385818; doi:10.3390/metabo13070868)
Supplement: Supplementary file 1 [file metabolites-13-00868-s001.zip › Table S2.pdf]

Table S2 The The concentration and A260/A280 ratios of total RNA of samples

Sample	F	SW	T3	CK3
Concentration (ng/μl)	675.5	630.2	750.3	798.3
A260/A280	2.09	2.08	2.07	2.07

F: fresh tea leaves; SW: solar-withered leaves; T3: turn-over treatment for three times; CK3: control group without turn-over treatment for three times.
