# Supplementary material for: The Dynamic Change in Aromatic Compounds and Their Relationship with CsAAAT Genes during the Post-Harvest Process of Oolong Tea
Source: Metabolites. 2023 Jul 20;13(7):868. doi: 10.3390/metabo13070868 (PMC10385818; doi:10.3390/metabo13070868)
Supplement: Supplementary file 1 [file metabolites-13-00868-s001.zip › Table S3.pdf]

Table S3 The Nucleic acid sequence of 16 candidate *CsAAAT* genes

Transcript	Number	Nucleic acid sequence(5'-3')
		ATGTTGTTGGAGAAAAGAATTTGTTGTTTAGCTTTAAGGATCATCATCATATTG
		CTAATTAGGAAGCTCAATTTTTGGGACTCTTTGGTCTTCTCAATTTTTGCAGAC
		CATACCATACCAGAGGTACACGGAACAGTGAGATACAGAGACAGAGATTTGG
		AATTTGGAGCCAAGATTTTGAATAATGGAAGTAGCCAACAATTGGAAACGCC
		GACGAACATCACAATCAAAGGCATTCTGGGTCTGTTGATGGCCAACATCGATT
		CCAACGACGAGAGGCGAATTATTTCTCTGGGTATGGGAGACCCATCTGCCTAC
		TCTTGCTTCCACACCACTGTTGTTGCTGAAGAAGCTGTGGTCGATTCTCTTCG
		CTCTGAGAAGTTCAATGGCTACTCTCCCACAGTCGGTCTTCCTCAAACCAGAA
		CTGCAATTGCCGAATATTTGTCACGTGATCTCCCTTACAAGTTATCACCCGACG
		ATGCTTTTGTCACATCTGGTTGCACTCAAGCTATTGATGTAGCATTGTCAATGT
		TAGCTCGCCCGGGAGTGAATATTTTGCTTCCAAGGCCAGGGTTCCCAATCTAC
		GAACTTTGTGCTGCTTTTAGACATCTTGAAGTTCGGCATTTTGATCTTCTTCCC
		GAAAAGGGTTGGGAAGTTGATCTTGATGCTGTCGAAGCTCTTGCAGACCATA
Unigene22082_All	*CsAAAT1-1*	ACACTGCTGCAATGGTAATCATAAATCCTGGGAATCCATGTGGCAACGTCTATA
		CTTATCAACATTTGAAGAAGATTGCTGAAACTGCTAAAAGGCTGAAAATTCTT
		GTAATTGCTGATGAAGTTTATGGCCACCTTGCTTTTGGGGCTAATCCTTTTGTG
		CCGATGGGAGTTTTTGGTTCAGTTGTTCCAGTTCTCACGCTTGGCTCTTTATCA
		AAGCGATGGATAGTCCCCGGTTGGCGACTTGGTTGGTTTGTGATAAATGATCC
		AAATGACATCTTCAGAAACCCAAAGATTGTTGAGCGGCTTAAGAAGTATTTTG
		ATATTCTTGGGGGTCCTTCGACATTTATACAGGCAGCAGTCCCGCGCATTCTTC
		AGCAAACTGAAGGCGTATTCTTTACAAAGACCATTAATATACTAAAGCAGACC
		TCTGATATTTGTTGTGAAAGAATAAAGGAGATTCCTTGCATTTCATGCCCATAT
		AAACCAGTGGGATCCATGGCTGTTATGATGAAACTGAATGTTTCCCTCCTCAA
		AGACATTAGCGATGACATTGACTTTTGCTTCAAACTGGCCAAGGAGGAATCTG
		TGATCATTCTTCCAGGACTTGCTGTGGGTATGAAAGATTGGATTCGCATCACTT
		TCGCGGTTGAACCATCTTCACTTGAAGAAGCTTTCGGGAGGGTGAAGTCTTT
		CTATCACCGACACTCCAATCAGCAAATAGAATACTAA
		ATGGAAAATGGAGGGAAGAAATTGAATAATGGAAGTAGCCAACAATTCGAAA
		CGCCGACGAACATCACAATCAAAGGCATTCTGGGTCTGTTGATGGCCAACATC
		GATTCCAACGACGAGAGGCGAATTATTTCTCTGGGTATGGGAGACCCATCTGC
		CTACTCTTGCTTCCACACCACTGTTGTTGCTGAAGAAGCTGTGGTCGATTCTC
		TTCGCTCTGAGAAGTTCAATGGCTACTCTCCCACAGTCGGTCTTCCTCAAACC
		AGAACTGCAATTGCCGAATATTTGTCACGTGATCTCCCTTACAAGTTATCACCC
		GACGATGCTTTTGTCACATCTGGTTGCACTCAAGCTATTGATGTAGCATTGTCA
Unigene17062_All	*CsAAAT1-2*	ATGTTAGCTCGCCCGGGAGTGAATATTTTGCTTCCAAGGCCAGGGTTCCCAAT
		CTACGAACTTTGTGCTGCTTTTAGACATCTTGAAGTTCGGCATTTTGATCTTCT
		TCCCGAAAAGGGTTGGGAAGTTGATCTTGATGCTGTCGAAGCTCTTGCAGAC
		CATAACACTGCTGCAATGGTAATCATAAATCCTGGGAATCCATGTGGCAATGTC
		TATACTTATCAACATTTGAAGAAGATTGCTGAAACTGCTAAAAGGCTGAAAAT
		TCCTGTAATTGCTGATGAAGTTTATGGCCACCTTGCTTTTGGGGCTAATCCTTT
		TGTGCCGATGGGAGTTTTTGGTTCAGTTGTTCCAGTTCTCACGCTTGGCTCTTT
		ATCAAAGCGATGGATAGTCCCCGGTTGGCGACTTGGTTGGTTTGTGATAAATG

		ATCCAAATGACATCTTCAGAAACCCAAAGATTGTTGAGCGGCTTAAGAAGTAT<br>TTTGATATTCTTGGGGGTCCTTCGACATTTATACAGGCAGCAGTCCCGCGCATT<br>CTTCAGCAAACTGAAGACGTATTCTTTACAAAGACCATTAATATACTAAAGCA<br>GACCTCTGATATTTGTTGTGAAAGAATAAAGGAGATTCCTTGCATTTCATGCCC<br>ATATAAACCAGTGGGATCCATGGCTGTTATGATGAAACTGAATGTTTCCCTCCT<br>CAAAGACATTAGCGATGACATTGACTTTTGCTTCAAACTGGCCAAGGAGGAAT<br>CTGTGATCATTCTTCCAGGACTTGCTGTGGGTATGAAAGATTGGATTCGCATCA<br>CTTTCGCGGTTGAACCATCTTCACTTGAAGAAGCTTTCGGGAGGGTGAAGTC<br>TTTCTATCACCGACACTCCAATCAGCAAATAGAATACTAA
Unigene13237_All	*CsAAAT1-3*	ATGTTGTTGGAGAAAAGAATTTGTTGTTTAGCTTTAAGGATCATCATCATATTG<br>CTAATTAGGAAGCTCAATTTTTGGGACTCTTTGGTCTTCTCAATTTTTGCAGAC<br>CATACCATACCAGAGCTACACGGAACAGTGAGATACAGAGACAGAGAAATGG<br>AAAATGGAGGGAAGAAATTGAATAATGGAAGTAGCCAACAATTCGAAACGCC<br>GACGAACATCACAATCAAAGGCATTCTGGGTGTGTTGATGGCCAACATCGATT<br>CCAACGACGAGAGGCGAATTATAACTCTGCCTATGCCAGACGGATCTGCCTAC<br>TCTTGCTTCCAGACCACTGTTGTTGCTGAAGAAGCTGTGGTCGATTCTCTTCG<br>CTCTGAGAAGTTCAATGGCTACTCTCCCACAGTCGGTCTTCCTCAAACCAGAA<br>CGGCAATTGCCGAATATTTGTCACGTGATCTCCCTTACAAGTTATCACCCGACG<br>ATGCTTTTGTCACATCTGGTTGCACTCAAGCTATTGATGTAGCATTGTCAATGT<br>TAGCTCGCCCGGGAGTGAATATTTTGCTTCCAAGGCCAGGGTTCCCAATCTAC<br>GAACTTTGTGCTGCTTTTAGACATCTTGAAGTTCGGCATTTTGATCTTCTTCCC<br>GAAAAGGGTTGGGAAGTTGATCTTGATGCTGTCGAAGCTCTTGCAGACCATA<br>ACACTGCTGCAATGGTAATCATAAATCCTGGGAATCCATGTGGCAATGTCTATA<br>CTTATCAACATTTGAAGAAGATTGCTGAAACTGCTAAAAGGCTGAAAATTCCT<br>GTAATTGCTGATGAAGTTTATGGCCACCTTGCTTTTGGGGCTAATCCTTTTGTG<br>CCGATGGGAGTTTTTGGTTCAGTTGTTCCAGTTCTCACGCTTGGCTCTTTATCA<br>AAGCGATGGATAGTCCCCGGTTGGCGACTTGGTTGGTTTGTGATAAATGATCC<br>AAATGACATCTTCAGAAACCCAAAGATTGTTGAGCGGCTTAAGAAGTATTTTG<br>ATATTCTTGGGGGTCCTTCGACATTTATACAGGCAGCAGTCCCGCGCATTCTTC<br>AGCAAACTGAAGGCGTATTCTTTACAAAGACCATTAATATACTAAAGCAGACC<br>TCTGATATTTGTTGTGAAAGAATAAAGGAGATTCCTTGCATTTCATGCCCATAT<br>AAACCAGTGGGATCCATGGCTGTTATGATGAAACTGAATGTTTCCCTCCTCAA<br>AGACATTAGCGATGACATTGACTTTTGCTTCAAACTGGCCAAGGAGGAATCTG<br>TGATCATTCTTCCAGGTGAGATACGAATCATCAATCGATCGCCTAATTAG
Unigene65900_All	*CsAAAT1-4*	ATGTTGTTGGAGAAAAGAATTTGTTGTTTAGCTTTAAGGATCATCATCATATTG<br>CTAATTAGGAAGCTCAATTTTTGGGACTCTTTGGTCTTCTCAATTTTTGCAGAC<br>CATACCATACCAGAGCTACACGGAACAGTGAGATACAGAGACAGAGAAATGG<br>AAAATGGAGGGAAGAAATTGAATAATGGAAGTAGCCAACAATTCGAAACGCC<br>GACGAACATCACAATCAAAGGCATTCTGGGTCTGTTGATGGCCAACATCGATT<br>CCAACGACGAGAGGCGAATTATTTCTCTGGGTATGGGAGACCCATCTGCCTAC<br>TCTTGCTTCCACACCACTGTTGTTGCTGAAGAAGCTGTGGTCGATTCTCTTCG<br>CTCTGAGAAGTTCAATGGCTACTCTCCCACAGTCGGTCTTCCTCAAACCAGAA<br>CGGCAATTGCCGAATATTTGTCACGTGATCTCCCTTACAAGTTATCACCCGACG<br>ATGCTTTTGTCACATCTGGTTGCACTCAAGCTATTGATGTAGCATTGTCAATGT

		TAGCTCGCCCGGGAGTGAATATTTTGCTTCCAAGGCCAGGGTTCCCAATCTAC<br>GAACTTTGTGCTGCTTTTAGACATCTTGAAGTTCGGCATTTTGATCTTCTTCCC<br>GAAAAGGGTTGGGAAGTTGATCTTGATGCTGTCGAAGCTCTTGCAGACCATA<br>ACACTGCTGCAATGGTAATCATAAATCCTGGGAATCCATGTGGCAATGTCTATA<br>CTTATCAACATTTGAAGAAGATTGCTGAAACTGCTAAAAGGCTGAAAATTCCT<br>GTAATTGCTGATGAAGTTTATGGCCACCTTGCTTTTGGGGCTAATCCTTTTGTG<br>CCGATGGGAGTTTTTGGTTCAGTTGTTCCAGTTCTCACGCTTGGCTCTTTATCA<br>AAGCGATGGATAGTCCCCGGTTGGCGACTTGGTTGGTTTGTGATAAATGATCC<br>AAATGACATCTTCAGAAACCCAAAGATTGTTGAGCGGCTTAAGAAGTATTTTG<br>ATATTCTTGGGGGTCCTTCGACATTTATACAGAATTCTGAGGAATAA
Unigene71295_All	*CsAAAT1-5*	ATGGAAAATGGAGGGAAGAAATTGAATAATGGAAGTAGCCAACAATTCGAAA<br>CGCCGACGAACATCACAATCAAAGGCATTCTGGGTCTGTTGATGGCCAACATC<br>GATTCCAACGACGAGAGGCGAATGATTTCTCTGGGTATGGGAGACCCATCTGC<br>CTACTCTTGCTTCCACACCACTGTTGTTGCTGAAGAAGCTGTGGTCGATTCTC<br>TTCGCTCTGAGAAGTTCAATGGCTACTCTCCCACAGTCGGTCTTCCTCAAACC<br>AGAACTGCAATTGCCGAATATTTGTCACGTGATCTCCCTTACAAGTTATCACCC<br>GACGATGCTTTTGTCACATCTGGTTGCACTCAAGCTATTGATGTAGCATTGTCA<br>ATGTTAGCTCGCCCGGGAGTGAATATTTTGCTTCCAAGGCCAGGGTTCCCAAT<br>CTACGAACTTTGTGCTGCTTTTAGACATCTTGAAGTTCGGCATTTTGATCTTCT<br>TCCCGAAAAGGGTTGGGAAGTTGATCTTGATGCTGTCGAAGCTCTTGCAGAC<br>CATAACACTGCTGCAATGGTAATCATAAATCCTGGGAATCCATGTGGCAATGTC<br>TATACTTATCAACATTTGAAGAAGATTGCTGAAACTGCTAAAAGGCTGAAAAT<br>TCCTGTAATTGCTGATGAAGTTTATGGCCACCTTGCTTTTGGGGCTAATCCTTT<br>TGTGCCGATGGGAGTTTTTGGTTCAGTTGTTCCAGTTCTCACGCTTGGCTCTTT<br>ATCAAAGCGATGGATAGTCCCCGGTTGGCGACTTGGTTGGTTTGTGATAAATG<br>ATCCAAATGACATCTTCAGAAACCCAAAGATTGTTGAGCGGCTTAAGAAGTAT<br>TTTGATATTCTTGGGGGTCCTTCGACATTTATACAGAATTCTGAGGAATAA
Unigene65894_All	*CsAAAT1-6*	ATGTTGTTGGAGAAAAGAATTTGTTGTTTAGCTTTAAGGATCATCATCATATTG<br>CTAATTAGGAAGCTCAATTTTTGGGACTCTTTGGTCTTCTCAATTTTTGCAGAC<br>CATACCATACCAGAGCTACACGGAACAGTGAGATACAGAGACAGAGAAATGG<br>AAAATGGAGGGAAGAAATTGAATAATGGAAGTAGCCAACAATTCGAAACGCC<br>GACGAACATCACAATCAAAGGCATTCTGGGTCTGTTGATGGCCAACATCGATT<br>CCAACGACGAGAGGCGAATTATTTCTCTGGGTATGGGAGACCCATCTGCCTAC<br>TCTTGCTTCCACACCACTGTTGTTGCTGAAGAAGCTGTGGTCGATTCTCTTCG<br>CTCTGAGAAGTTCAATGGCTACTCTCCCACAGTCGGTCTTCCTCAAACCAGAA<br>CGGCAATTGCCGAATATTTGTCACGTGATCTCCCTTACAAGTTATCACCCGACG<br>ATGCTTTTGTCACATCTGGTTGCACTCAAGCTATTGATGTAGCATTGTCAATGT<br>TAGCTCGCCCGGGAGTGAATATTTTGCTTCCAAGGCCAGGGTTCCCAATCTAC<br>GAACTTTGTGCTGCTTTTAGACATCTTGAAGTTCGGCATTTTGATCTTCTTCCC<br>GAAAAGGGTTGGGAAGTTGATCTTGATGCTGTCGAAGCTCTTGCAGACCATA<br>ACACTGCTGCAATGGTAATCATAAATCCTGGGAATCCATGTGGCAATGTCTATA<br>CTTATCAACATTTGAAGAAGATTGCTGAAACTGCAAAAAGGCTGAAAATTCTT<br>GTAATTGCTGATGAAGTTTATGGCCACCTTGCTTTTGGGGCTAATCCTTTTGTG<br>TCGATTGGAGTCTTTGGATCGGTTGTTCCAGTTCTCACGCTTGGGTCTTTATCA

		AAGCGATGGATAGTCCCCGGTTGGCGACTTGGTTGGTTTGTGATAAATGATCC<br>AAATGACATCTTCAGAAACCCAAAGATTGTTGAGCGGCTTAAGAAGTATTTTG<br>ATATTCTTGGGGGTCCTTCGACATTTATACAGAATTCTGAGGAATAA
Unigene3903_All	*CsAAAT1-7*	ATGGAAAATGGAGGGAAGAAATTGAATAATGGAAGTAGCCAACAATTCGAAA<br>CGCCGACGAACATCACAATCAAAGGCATTCTGGGTCTGTTGATGGCCAACATC<br>GATTCCAACGACGAGAGGCGAATTATTTCTCTGGGTATGGGAGACCCATCTGC<br>CTACTCTTGCTTCCACACCACTGTTGTTGCTGAAGAAGCTGTGGTCGATTCTC<br>TTCGCTCTGAGAAGTTCAATGGCTACTCTCCCACAGTCGGTCTTCCTCAAACC<br>AGAACTGCAATTGCCGAATATTTGTCACGTGATCTCCCTTACAAGTTATCACCC<br>GACGATGCTTTTGTCACATCTGGTTGCACTCAAGCTATTGATGTAGCATTGTCA<br>ATGTTAGCTCGCCCGGGAGTGAATATTTTGCTTCCAAGGCCAGGGTTCCCAAT<br>CTACGAACTTTGTGCTGCTTTTAGACATCTTGAAGTTCGGCATTTTGATCTTCT<br>TCCCGAAAAGGGTTGGGAAGTTGATCTTGATGCTGTCGAAGCTCTTGCAGAC<br>CATAACACTGCTGCAATGGTAATCATAAATCCTGGGAATCCATGTGGCAACGT<br>CTATACTTATCAACATTTGAAGAAGATTGCTGAAACTGCTAAAAGGCTGAAAA<br>TTCCTGTAATTGCTGATGAAGTTTATGGCCACCTTGCTTTTGGGGCTAATCCTT<br>TTGTGCCGATGGGAGTTTTTGGTTCAGTTGTTCCAGTTCTCACGCTTGGCTCTT<br>TATCAAAGCGATGGATAGTCCCCGGTTGGCGACTTGGTTGGTTTGTGATAAAT<br>GATCCAAGTGACTTCTTCAAAAACCCCAAGGTTTGCTTTGTGTACTTTCTCTT<br>CTGGTAA
Unigene39034_All	*CsAAAT1-8*	TGTTGTTTAGCTTTAAGGATCATCATCATATTGCTAATTAGGAAGCTCAATTTTT<br>GGGACTCTTTGGTCTTCTCAATTTTTGCAGACCATACCATACCAGAGCTACAC<br>GGAACAGTGAGATACAGAGACAGAGAAATGGAAAATGGAGGGAAGAAATTG<br>AATAATGGAAGTAGCCAACAATTCGAAACGCCGACGAACATCACAATCAAAG<br>GCATTCTGGGTCTGTTGATGGCCAACATCGATTCCAACGACGAGAGGCGAATT<br>ATTTCTCTGGGTATGGGAGACCCATCTGCCTACTCTTGCTTCCACACCACTGTT<br>GTTGCTGAAGAAGCTGTGGTCGATTCTCTTCGCTCTGAGAAGTTCAATGGCTA<br>CTCTCCCACAGTCGGTCTTCCTCAAACCAGAACTGCAATTGCCGAATATTTGT<br>CACGTGATCTCCCTTACAAGTTATCACCCGACGATGCTTTTGTCACATCTGGTT<br>GCACTCAAGCTATTGATGTAGCATTGTCAATGTTAGCTCGCCCGGGAGTGAATA<br>TTTTGCTTCCAAGGCCAGGGTTCCCAATCTACGAACTTTGTGCTGCTTTTAGA<br>CATCTTGAAGTTCGGCATTTTGATCTTCTTCCCGAAAAGGGTTGGGAAGTTGA<br>TCTTGATGCTGTCGAAGCTCTTGCAGACCATAACACTGCTGCAATGGTAATCAT<br>AAATCCTGGGAATCCATGTGGCAACGTCTATACTTATCAACATTTGAAGAAGAT<br>TGCTGAAACTGCTAAAAGGCTGAAAATTCCTGTAATTGCTGATGAAGTTTATG<br>GCCACCTTGCTTTTGGGGCTAATCCTTTTGTGCCGATGGGAGTTTTTGGTTCAG<br>TTGTTCCAGTTCTCACGCTTGGCTCTTTATCAAAGCGATGGATAGTCCCCGGTT<br>GGCGACTTGGTTGGTTTGTGATAAATGATCCAAGTGACTTCTTCAAAAACCCC<br>AAGGTTTGCTTTGTGTACTTTCTCTTCTGGTAA
Unigene63900_All	*CsAAAT1-9*	ATGGGAGTTTTTGGTTCAGTTGTTCCAGTTCTCACGCTTGGCTCTTTATCAAAG<br>CGATGGATAGTCCCCGGTTGGCGACTTGGTTGGTTTGTGATAAATGATCCAAA<br>TGACATCTTCAGAAACCCAAAGATTGTTGAGCGGCTTAAGAAGTATTTTGATA<br>TTCTTGGGGGTCCTTCGACATTTATACAGGCAGCAGTCCCGCGCATTCTTCAG<br>CAAACTGAAGACGTATTCTTTACAAAGACCATTAATATACTAAAGCAGACCTC

		TGATATTTGTTGTGAAAGAATAAAGGAGATTCCTTGCATTTCATGCCCATATAA<br>ACCAGTGGGATCCATGGCTGTTATGATGAAACTGAATGTTTCCCTCCTCAAAG<br>ACATTAGCGATCACATTGACTTAAGCTTCATTCTGGCCAAGGAGGAATCTGTG<br>ATCATTCTTCCAGGACTTGCTGTGCCTATGATTGATTGGATTCGCATCACTAAC<br>GCGGTTGAACCATCTTCACTTGAAGAAGGTTTCGGGAGGGTGAAGTCTTTCTA<br>TCACCGACACTCCAATCAGCAAATAGAATACTAA
CL11783.Contig7_All	*CsAAAT2-1*	ATGGAGAACGGATCATCGACGGCGGTGAGATGGGGTTTCCAAGGGAACAAA<br>GGGCTGAGCACGGCGTCGGCGATCACCATTAGAGGAATTCTGAACGAGGTTA<br>CGGAAAACATCGACAAAGCAGACCCTAGGCCCATCATTCCTCTCGGCCACGG<br>CGACCCCTCTGCTTTTCCCTGCTTTCGGACCACTCCTTTCGCCGAGGACGCCA<br>TCGCCGACGCCGTCCGATCCGCCAAATTCAACGGTTATTCTCCTACCGTCGGTA<br>TTCTTCCGGCTCGAAGGGCCATAGCAGAGTACCTCTCAAAAGATCTTCCATAC<br>AAGTTGTCACCAGACGATGTTTATTTGACAATTGGATGTACACAAGCAATTGA<br>AGTTATGTTAACAGTTCTTGCTCGTCCATATGCTTACATTCTGCTGGCAAGACC<br>AGGCTTGGCACTCTATGAACCGCGTGCTGCTTATAGCCATCTTGAAGTCCGCC<br>ACTATGATCTTCTACCAGAGAAAGGTTGGGAGGTAGATCTTGATGCAGTTGAA<br>GCTCTAGCAGATGAAAATACTGTCGCTATGGCTATTATTAATCCTGGCAATCCT<br>TGTGGGAATGTCTTTACATACCAACATTTAAAGCAGGTTGCTAAGACTGCAAG<br>AAAGCTTGGAATATTAGTGATTGCTGATGAAGTTTATGATCATCTTACTTTTGG<br>GAGTAACCCATTCGTGCCCATGGGAGTCTTTGGATCAATTGCACCTGTTATTAC<br>AGTTGGGTCTATATCCAAGAGGTGGATTGTTCCAGGTTGGCGACTTGGTTGGC<br>TTGTCACAAATGATCCCAATGGCATCCTTGCAAAATATGGGATAGTTGACTGCA<br>TTAAGGGCTTTCTAAATATTTCCGCTGACCCGGCAACTTTCATTCAGGGGGCA<br>ATTCCTCAAATCCTTGAGAAAACGAAAGATGATTTCTTTTCCAAAATTGTTAAT<br>GTACTGAGAGAAGCTGCAGACATTTGCTATTATGGAATTAAAGAGATCCCTTG<br>CATTTCTTGCCCAAGCAAACCTGAAGGATCTATGTTTGTAATGGTGAAACTAA<br>ATCTGTCACTCCTGGAAGACATTAAAGATGATATTGACTTCTCTTTCAAGCTGG<br>CAAAGGAGGAGTCTGTGATTGTTCTACCAGGGGTGGCTGTAGGACTAAAGAA<br>TTGGCTCCGCATAACATTTGCCATTGAGCCATCATCTCTTGAAGAAGGCCTGG<br>GGAGAATAAAAGCTTTCTACCAAAGGCATGCTAAGAAGCAATAA
CL11783.Contig1_All	*CsAAAT2-2*	ATGGGGCTAAATAGCTCCCAAGAAACTGCTTCCGCTGGGCTCAATCCACCGCC<br>TATGGTTCTAGCCGAGCTGGCTTCTTCGGCGCCGACCAGCCGACCCTGGAGA<br>GACACCCATGCAAGCTGTCTCGCTGCTCTCACGGGCCGATTCGGCCTTGTAAA<br>ATGGCTCGAGAGCCGGCGCGGGGAGGGAGAGACTGAAGGCGAGAATGGCGT<br>CAGAGTGACGATAGATGGCGCCGCAAAAGCAGCCCCGAAGCCGCTCTCCGGC<br>TCTTCTTCATCCATGGACACACACACAGAGAGAGAGAGAGAGAGAAGATGGAG<br>AACGGATCATCGACGGCGGTGAGATGGGGTTTCCAAGGGAACAAAGGGCTG<br>AGCACGGCGTCGGCGATCACCATTAGAGGAATTCTGAACGAGGTTACGGAAA<br>ACATCGACAAAGCAGACCCTAGGCCCATCATTCCTCTCGGCCACGGCGACCC<br>CTCTGCTTTTCCCTGCTTTCGGACCACTCCTTTCGCCGAGGACGCCATCGCCG<br>ACGCCGTCCGATCCGCCAAATTCAACGGTTATTCTCCTACCGTCGGTATTCTTC<br>CGGCTCGAAGGGCCATAGCAGAGTACCTCTCAAAAGATCTTCCATACAAGTTG<br>TCACCAGACGATGTTTATTTGACAATTGGATGTACACAAGCAATTGAAGTTATG<br>TTAACAGTTCTTGCTCGTCCAAATGCAAACATTCTGCTCCCAAGACCAGGCTT

		CCCACTCTATGAAGCCCGTGCTGCATTTAGCCATCTTGAAGTCCGCCACTATGA<br>TCTTCTTCCAGAGAAAGGTTGGGAGGTAGATCTTGATGCAGTTGAAGCTCTAG<br>CAGATGAAAATACTGTCGCTATGGCTATTATTAATCCTGGCAATCCTTGTGGGA<br>ATGTCTTTACATACCAACATTTAAAGCAGGTTGCTAAGACTGCAAGAAAGCTT<br>GGAATATTAGTGATTGCTGATGAAGTTTATGATCATCTTACTTTTGGGAGTAAC<br>CCATTCGTGCCCATGGGAGTCTTTGGATCAATTGCACCTGTTATTACAGTTGGG<br>TCTATATCCAAGAGGTGGATTGTTCCAGGTTGGCGACTTGGTTGGCTTGTCAC<br>AAATGATCCCAATGGCATCCTTGCAAAATATGGGATAGTTGACTGCATTAAGG<br>GCTTTCTAAATATTTCCGCTGACCCGGCAACTTTCATTCAGGGGGCAATTCCTC<br>AAATCCTTGAGAAAACGAAAGATGATTTCTTTTCCAAAATTGTTAATGTACTG<br>AGAGAAGCTGCAGACATTTGCTATTATGGAATTAAAGAGATCCCTTGCATTTCT<br>TGCCCAAGCAAACCTGAAGGATCTATGTTTGTAATGGTGAAACTAAATCTGTC<br>ACTCCTGGAAGACATTAAAGATGATATTGACTTCTCTTTCAAGCTGGCAAAGG<br>AGGAGTCTGTGATTGTTCTACCAGGGGTGGCTGTAGGACTAAAGAATTGGCTC<br>CGCATAACATTTGCCATTGAGCCATCATCTCTTGAAGAAGGCCTGGGGAGAAT<br>AAAAGCTTTCTACCAAAGGCATGCTAAGAAGCAATAA
CL11783.Contig12_All	*CsAAAT2-3*	CAAGGGAACAAAGGGCTGAGCACGGCGTCGGCGATCACCATTAGAGGAATTC<br>TGAACGAGGTTACGGAAAACATCGACAAAGCAGACCCTAGGCCCATCATTCC<br>TCTCGGCCACGGCGACCCCTCTGCTTTTCCCTGCTTTCGGACCACTCCTTTCG<br>CCGAGGACGCCATCGCCGACGCCGTCCGATCCGCCAAATTCAACGGTTATTCT<br>CCTACCGTCGGTATTCTTCCGGCTCGAAGGGCCATAGCAGAGTACCTCTCAAA<br>AGATCTTCCATACAAGTTGTCACCAGACGATGTTTATTTGACAATTGGATGTAC<br>ACAAGCAATTGAAGTTATGTTAACAGTTCTTGCTCGTCCAAATGCAAACATTC<br>TGCTCCCAAGACCAGGCATGGCACTCTATGAACCGCGTGCTGCTTATAGCCAT<br>CTTGAAGTCCGCCACTATGATCTTCTACCAGAGAAAGGTTGGGAGGTAGATCT<br>TGATGCAGTTGAAGCTCTAGCAGATGAAAATACTGTCGCTATGGCTATTATTAA<br>TCCTGGCAATCCTTGTGGGAATGTCTTTACATACCAACATTTAAAGCAGGTTGC<br>TAAGACTGCAAGAAAGCTTGGAATATTAGTGATTGCTGATGAAGTTTATGATCA<br>TCTTACTTTTGGGAGTAACCCATTCGTGCCCATGGGAGTCTTTGGATCAATTGC<br>ACCTGTTATTACAGTTGGGTCTATATCCAAGAGGTGGATTGTTCCAGGTTGGCG<br>ACTTGGTTGGCTTGTCACAAATGATCCCAATGGCATCCTTGCAAAATATGGGAT<br>AGTTGACTGCATTAAGGGCTTTCTAAATATTTCCGCTGACCCGGCAACTTTCAT<br>TCAGGGGGCAATTCCTCAAATCCTTGAGAAAACGAAAGATGATTTCTTTTCCA<br>AAATTGTTAATGTACTGAGAGAAGCTGCAGACATTTGCTATTATGGAATTAAAG<br>AGATCCCTTGCATTTCTTGCCCAAGCAAACCTGAAGGATCTATGTTTGTAATGG<br>TGAAACTAAATCTGTCACTCCTGGAAGACATTAAAGATGATATTGACTTCTCTT<br>TCAAGCTGGCAAAGGAGGAGTCTGTGATTGTTCTACCAGGGGTGGCTGTAGG<br>ACTAAAGAATTGGCTCCGCATAACATTTGCCATTGAGCCATCATCTCTTGAAG<br>AAGGCCTGGGGAGAATAAAAGCTTTCTACCAAAGGCATGCTAAGAAGCAATA<br>A
CL11783.Contig2_All	*CsAAAT2-4*	ATGGAGAACGGATCATCGACGGCGGTGAGATGGGGTTTCCAAGGGAACAAA<br>GGGCTGAGCACGGCGTCGGCGATCACCATTAGAGGAATTCTGAACGAGGTTA<br>CGGAAAACATCGACAAAGCAGACCCTAGGCCCATCATTCCTCTCGGCCACGG<br>CGACCCCTCTGCTTTTCCCTGCTTTCGGACCACTCCTTTCGCCGAGGACGCCA

		TCGCCGACGCCGTCCGATCCGCCAAATTCAACGGTTATTCTCCTACCGTCGGTA<br>TTCTTCCGGCTCGAAGGGCCATAGCAGAGTACCTCTCAAAAGATCTTCCATAC<br>AAGTTGTCACCAGACGATGTTTATTTGACAATTGGATGTACACAAGCAATTGA<br>AGTTATGTTAACAGTTCTTGCTCGTCCAAATGCAAACATTCTGCTCCCAAGAC<br>CAGGCTTCCCACTCTATGAAGCCCGTGCTGCATTTAGCCATCTTGAAGTCCGC<br>CACTATGATCTTCTTCCAGAGAAAGGTTGGGAGGTAGATCTTGATGCAGTTGA<br>AGCTCTAGCAGATGAAAATACTGTCGCTATGGCTATTATTAATCCTGGCAATCC<br>TTGTGGGAATGTCTTTACATACCAACATTTAAAGCAGGTTGCTAAGACTGCAA<br>GAAAGCTTGGAATATTAGTGATTGCTGATGAAGTTTATGATCATCTTACTTTTG<br>GGAGTAACCCATTCGTGCCCATGGGAGTCTTTGGATCAATTGCACCTGTTATTA<br>CAGTTGGGTCTATATCCAAGAGGTGGATTGTTCCAGGTTGGCGACTTGGTTGG<br>CTTGTCACAAATGATCCCAATGGCATCCTTGCAAAATATGGGATAGTTGACTGC<br>ATTAAGGGCTTTCTAAATATTTCCGCTGACCCGGCAACTTTCATTCAGGGGGC<br>AATTCCTCAAATCCTTGAGAAAACGAAAGATGATTTCTTTTCCAAAATTGTTAA<br>TGTACTGAGAGAAGCTGCAGACATTTGCTATTATGGAATTAAAGAGATCCCTT<br>GCATTTCTTGCCCAAGCAAACCTGAAGGATCTATGTTTGTAATGGTGAAACTA<br>AATCTGTCACTCCTGGAAGACATTAAAGATGATATTGACTTCTCTTTCAAGCTG<br>GCAAAGGAGGAGTCTGTGATTGTTCTACCAGGTAAGAAGCAAACAAATTAA
CL11783.Contig5_All	*CsAAAT2-5*	ACAAACCCTTCTCTCTCTCTCTTTCTCTTTCTCTTTCTCTTTCTCTCTCATACAC<br>ACACACACACGCGCACACACACACAGAGAGAGAGAGAGAGAGAAGATGGAGAAC<br>GGATCATCGACGGCGGTGAGATGGGGTTTCCAAGGGAACAAAGGGCTGAGC<br>ACGGCGTCGGCGATCACCATTAGAGGAATTCTGAACGAGGTTACGGAAAACA<br>TCGACAAAGCAGACCCTAGGCCCATCATTCCTCTCGGCCACGGCGACCCCTCT<br>GCTTTTCCCTGCTTTCGGACCACTCCTTTCGCCGAGGACGCCATCGCCGACGC<br>CGTCCGATCCGCCAAATTCAACGGTTATTCTCCTACCGTCGGTATTCTTCCGGC<br>TCGAAGGGCCATAGCAGAGTACCTCTCAAAAGATCTTCCATACAAGTTGTCAC<br>CAGACGATGTTTATTTGACAATTGGATGTACACAAGCAATTGAAGTTATGTTAA<br>CAGTTCTTGCTCGTCCAAATGCAAACATTCTGCTCCCAAGACCAGGCTTCCCA<br>CTCTATGAAGCCCGTGCTGCATTTAGCCATCTTGAAGTCCGCCACTATGATCTT<br>CTTCCAGAGAAAGGTTGGGAGGTAGATCTTGATGCAGTTGAAGCTCTAGCAG<br>ATGAAAATACTGTCGCTATGGCTATTATTAATCCTGGCAATCCTTGTGGGAATG<br>TCTTTACATACCAACATTTAAAGCAGGTTGCTAAGACTGCAAGAAAGCTTGGA<br>ATATTAGTGATTGCTGATGAAGTTTATGATCATCTTACTTTTGGGAGTAACCCAT<br>TCGTGCCCATGGGAGTCTTTGGATCAATTGCACCTGTTATTACAGTTGGGTCTA<br>TATCCAAGAGGTGGATTGTTCCAGGTTGGCGACTTGGTTGGCTTGTCACAAAT<br>GATCCCAATGGCATCCTTGCAAAATATGGGATAGTTGACTGCATTAAGGGCTTT<br>CTAAATATTTCCGCTGACCCGGCAACTTTCATTCAGGGGGCAATTCCTCAAATC<br>CTTGAGAAAACGAAAGATGATTTCTTTTCCAAAATTGTTAATGTACTGAGAGA<br>AGCTGCAGACATTTGCTATTATGGAATTAAAGAGATCCCTTGCATTTCTTGCCC<br>AAGCAAACCTGAAGGATCTATGTTTGTAATGGTAAGGATGTAG
CL11783.Contig8_All	*CsAAAT2-6*	ACACAAACCCTTCTCTCTCTCTCTCTCTTTCTCTTTCTCTTTCTCTTTCTCTCTC<br>ATACACACACACACACGCGCTCACACACAGAGAGAGAGAGAGAGAGAAGATGG<br>AGAACGGATCATCGACGGCGGTGAGATCCGGTTACCAAGGGAACAAAGGGCT<br>GAGCACGGCGTCGGCGATCACCATTAGAGGAATTCTGAACGAGGTTATGGAG

		AACATCGACAAAGCAGACCCTAGGCCCATCATTCCTCTCGGCCACGGCGACC<br>CCTCTGCTTTTCCCTGCTTTCGGACCACTCCTTTCGCCGAGGACGCCATCGCC<br>GACGCCGTCCGATCCGCCAAATTCAACGGTTATTCTCCTACCGTCGGTATTCTT<br>CCGGCTCGAAGGGCCATAGCAGAGTACCTCTCAAAAGATCTTCCATACAAGTT<br>GTCACCAGACGATGTTTATTTGACAATTGGATGTACACAAGCAATTGAAGTTAT<br>GTTAACAGTTCTTGCTCGTCCAAATGCAAACATTCTGCTCCCAAGACCAGGCT<br>TCCCACTCTATGAAGCCCGTGCTGCATTTAGCCATCTTGAAGTCCGCCACTATG<br>ATCTTCTTCCAGAGAAAGGTTGGGAGGTAGATCTTGATGCAGTTGAAGCTCTA<br>GCAGATGAAAATACTGTCGCTATGGCTATTATTAATCCTGGCAATCCTTGTGGG<br>AATGTCTTTACATACCAACATTTAAAGCAGGTTGCTAAGACTGCAAGAAAGCT<br>TGGAATATTAGTGATTGCTGATGAAGTTTATGATCATCTTACTTTTGGGAGTAA<br>CCCATTCGTGCCCATGGGAGTCTTTGGATCAATTGCACCTGTTATTACAGTTGG<br>GTCTATATCCAAGAGGTGGATTGTTCCAGGTTGGCGACTTGGTTGGCTTGTCA<br>CAAATGATCCCAATGGCATCCTTGCAAAATATGGGATAGTTGACTGCATTAAGG<br>GCTTTCTAAATATTTCCGCTGACCCGGCAACTTTCATTCAGGTTTGTTGTTCAA<br>AATGCTTTTTCTGA
CL11783.Contig6_All	*CsAAAT2-7*	ACAAACCCTTCTCTCTCTCTCTTTCTCTTTCTCTTTCTCTTTCTCTCTCATACAC<br>ACACACACACGCGCACACACACAGAGAGAGAGAGAGAGAAGATGGAGAAC<br>GGATCATCGACGGCGGTGAGATGGGGTTTCCAAGGGAACAAAGGGCTGAGC<br>ACGGCGTCGGCGATCACCATTAGAGGAATTCTGAACGAGGTTACGGAAAACA<br>TCGACAAAGCAGACCCTAGGCCCATCATTCCTCTCGGCCACGGCGACCCCTCT<br>GCTTTTCCCTGCTTTCGGACCACTCCTTTCGCCGAGGACGCCATCGCCGACGC<br>CGTCCGATCCGCCAAATTCAACGGTTATTCTCCTACCGTCGGTATTCTTCCGGC<br>TCGAAGGGCCATAGCAGAGTACCTCTCAAAAGATCTTCCATACAAGTTGTCAC<br>CAGACGATGTTTATTTGACAATTGGATGTACACAAGCAATTGAAGTTATGTTAA<br>CAGTTCTTGCTCGTCCAAATGCAAACATTCTGCTCCCAAGACCAGGCTTCCCA<br>CTCTATGAAGCCCGTGCTGCATTTAGCCATCTTGAAGTCCGCCACTATGATCTT<br>CTTCCAGAGAAAGGTTGGGAGGTAGATCTTGATGCAGTTGAAGCTCTAGCAG<br>ATGAAAATACTGTCGCTATGGCTATTATTAATCCTGGCAATCCTTGTGGGAATG<br>TCTTTACATACCAACATTTAAAGCAGGTTGCTAAGACTGCAAGAAAGCTTGGA<br>ATATTAGTGATTGCTGATGAAGTTTATGATCATCTTACTTTTGGGAGTAACCCAT<br>TCGTGCCCATGGGAGTCTTTGGATCAATTGCACCTGTTATTACAGTTGGGTCTA<br>TATCCAAGAGGTGGATTGTTCCAGGTTGGCGACTTGGTTGGCTTGTCACAAAT<br>GATCCCAATGGCATCCTTGCAAAATATGGGGTTCTCTCTCTCTCTATCTCCCTC<br>TCTTCTCTCCCTCCCTCCCCTTGCATGTTTTCTCATGCCATAGTAGTGTCCCCCT<br>TATTTAGTCCAACAAGTATAGAACAAATTAACTCAATGTGA

The sequence highlighted in green represented the forward primer sequences(5’-3’); the sequence highlighted in yellow sequence represented reverse primer sequences(3’-5’).Underline indicated amplicon sequences(5’-3’) of qRT-PCR assay.
