# Supplementary material for: The Dynamic Change in Aromatic Compounds and Their Relationship with CsAAAT Genes during the Post-Harvest Process of Oolong Tea
Source: Metabolites. 2023 Jul 20;13(7):868. doi: 10.3390/metabo13070868 (PMC10385818; doi:10.3390/metabo13070868)
Supplement: Supplementary file 1 [file metabolites-13-00868-s001.zip › Table S4.pdf]

Table S4 Sensory evaluation results of raw oolong tea

	Appearance	Liquor colour	Aroma	Taste	Infused leaf
Rt	Wiry, yellow bloom	Golden yellow	Fragment and lasting，with flowery aroma	Mellow, fresh and brisk	Yellowish green and even with red even
Rc	Wiry, greenish yellow	Yellow with deep green	Clean and refreshing , less gentle flowery flavour	Heavy rather mellow with green and astringency	Yellowish green and even

Rt: raw oolong tea from T3 in experimental group; Rc: raw oolong tea from CK3 in control group; OVA: odor activity value
